# Year-round high abundances of the world’s smallest marine vertebrate (*Schindleria*) in the Red Sea and worldwide associations with lunar phases

**DOI:** 10.1038/s41598-021-93800-w

**Published:** 2021-07-12

**Authors:** Vanessa Robitzch, Victor Molina-Valdivia, Jaiber J. Solano-Iguaran, Mauricio F. Landaeta, Michael L. Berumen

**Affiliations:** 1 Red Sea Research Center, Division of Biological and Environmental Science and Engineering, King Abdullah University of Science and Technology, Thuwal, 23955 Saudi Arabia; 2 Facultad de Ciencias, Instituto de Ciencias Ambientales y Evolutivas, Universidad Austral de Chile, Casilla 567, Valdivia, Chile; 3 Laboratorio de Ictioplancton (LABITI), Facultad de Ciencias, Instituto de Biología, Universidad de Valparaíso, Valparaíso, Chile; 4 Universidad Católica de La Santísima Concepción, Programa de Magíster en Ecología Marina, Valparaíso, Chile; 5 Departamento de Salud Hidrobiológica, Instituto de Fomento Pesquero, Puerto Montt, Chile; 6 Centro de Observación Marino para Estudios de Riesgos del Ambiente Costero (COSTA-R), Universidad de Valparaíso, Viña del Mar, Chile

**Keywords:** Marine biology, Animal migration, Behavioural ecology, Population dynamics

## Abstract

Very little is known about the ecology and biology of the smallest marine vertebrates, fishes in the genus *Schindleria.* Even though over half of named *Schindleria* species have been identified in the Red Sea, the collection of only very few specimens has been documented. Here, we assessed abundance patterns of nearly two thousand Red Sea long dorsal fin (LDF) adults and found evidence for putative seasonal and spatial differences, likely related to differing habitat and environmental conditions. The highest abundances were outside local seasonal temperature extremes and decoupled from peaks of coral reef fish recruitment. We also found evidence for global trends in abundances related to lunar cycles using our Red Sea data and that from a recently published large collection of specimens from the DANA Expedition (1928–1930). The abundance of adult LDF *Schindleria* in relation to lunar phases differed significantly, with most *Schindleria* caught outside the full moon, and mostly during the new moon in the Red Sea and the 3rd quarter moon in the DANA collection. We further suggest that the abundances of *Schindleria* at coral reefs may be related to reproductive cycles and that these cycles may be timed with the moon as back-calculations of hatch dates from otoliths from the Red Sea significantly resulted after the new moon, making *Schindleria* the fastest-lived coral reef fish with the shortest generation times. *Schindleria* could be the most numerous coral reef fish in the world, for which we encourage increased research.

## Introduction

*Schindleria* (Giltay, 1934)^1^ is a small cryptic gobioid genus^2^ comprising the smallest vertebrates of the marine realm and the naturally youngest reproducing vertebrates on the planet^3^. Due to their cryptic nature and fragile bodies, apart from one photograph of an aggregation in the Hawaiian Islands taken by J. E. Randall documenting their residence over sand and rubble substrata during daytime^4^, they have never been sighted in their natural habitat elsewhere, nor has it been possible to maintain them in aquaria^5^. Hence, almost nothing is known about their biology, ecology, and exact distribution. Adult *Schindleria* are progenic, remain very small-sized (ca. 17 mm in average^6^), and have paedomorphic features (i.e., never undergoing metamorphosis and retaining a larval phenotype throughout their life^2^). Therefore, they are generally overlooked or mistaken for larval fishes^7,8^ and most studies are based on very few samples or report the sightings of some individuals as bycatch of other research purposes (mainly from light traps and planktonic tows)^9,10^. More recent sampling efforts^5,11^ and the inspection of museum collections from plankton tows for the presence of *Schindleria*^12^ have increased knowledge and attention on the presence and distribution of this genus, and high numbers of these fishes have increasingly been studied. Ahnelt and Sauberer (2020) provide a complete overview of all currently published *Schindleria* sightings and the data available on its distribution^12^. Apart from the Caribbean and the tropical Atlantic, the family seems to be common across coral reefs worldwide.

*Schindleria* can be found throughout the Indo-Pacific^11–13^ and are believed to be “lagoon-completers”, remaining for their entire lifecycle in shallow coral reef lagoons^14,15^. However, some specimens have also been collected in coastal waters around volcanic islands lacking lagoons (e.g., Easter Island^9^), as well as relatively distant from coral reefs and at depths of hundreds of meters^12,13,16–18^; but whether these findings are exceptions, strayed specimens, individuals advected by strong currents, or whether deeper oceanic environments are also part of *Schindleria’*s habitat, still remains unanswered and highlights the need for more targeted and comprehensive sampling efforts. Despite the lack of data on their ecology and biology mainly due to missing in situ observations of specimens, some authors suggest that *Schindleria* may live in burrows in the sandy bottoms of the coral reef matrix^14,19^ of which they come out at night, probably to feed and avoid visual predators. Other studies suggest that they retain a planktonic lifestyle, because most of their collections have been made with plankton tows in pelagic or neritic habitats^20–22^, but that they are yet in close association with coral reefs^10,11,15,23^; and whether the daytime aggregation (photographed over sand and rubble substrata in Hawaii^4^) is a common behavior or whether aggregations are seasonally induced, still remains unknown.

To date, very few *Schindleria* species have been described. From the seven named species^18^, four have been documented in the Red Sea and only a handful of specimens were collected of each of these species in the Red Sea^24–27^. Genetically, more than 40 different haplotypes have been identified in the eastern Indo-Pacific based on sequence data from the 16S mitochondrial rDNA gene region, suggesting that there may be a much higher number of species within the genus^5,11^. Morphologically, it has been very difficult to classify specimens or discriminate among haplotypes due to the lack of evident diagnostic features, likely because of their very simple body constructs^2^. As a result, the classification into two main types remains the most commonly used approach to describe the composition of *Schindleria* collections^12,28^: a long dorsal fin type (LDF; with the origin of the dorsal-fin anterior to the origin of the anal fin, by a minimum of six fin rays), which is also the most commonly sighted^12^; and a short dorsal fin type (SDF; with the origins of anal and dorsal fin more or less vertically aligned), which seems more rare and potentially comprises endemic species.

Our study assessed the abundances of adult LDF type specimens of *Schindleria* in the central Red Sea of Saudi Arabia, which is the only type so far documented in the region. We hypothesized that mature LDF *Schindleria* specimens may be aggregating in the shallow waters of coral reefs at night^10,15^ to reproduce and should thus be more abundant in the catches around the new moon or darker moon phases, during which their translucent bodies are less likely to be sighted by nocturnal predators. Many marine species show reproductive cycles linked to lunar patterns^29^. For this purpose, we collected specimens during the nights of an entire lunar month deploying LED light traps from mid-October to mid-November (in 2014) and assessed whether or not specimens were rather absent in the catches around the full moon. Additionally, we used the recently published data on the presence of *Schindleria* in the plankton tow collections from the DANA Expedition 1928–1930 (which sampled from Polynesia to southeast Africa^12^) and assessed whether or not abundances of adult *Schindleria* related to lunar phases is a global trend. To further support putative links between the life-cycles (i.e., biology and reproductive behavior) of *Schindleria* and lunar cycles, we assessed the ages of the adult *Schindleria* specimens from the Red Sea (by counting daily growth increments of their sagittal otoliths), inferred their hatch dates, and estimated the length of a putative reproductive cycle in these extremely short-lived fishes^3,9^. Lastly, our Red Sea reef sites comprised three reefs located along a cross-shelf gradient. Hence, we collected *Schindleria* deploying LED light traps around the new moons of twelve consecutive months (from February 2015 and to January 2016) to assess potential variations in their abundances related to spatio-temporal differences and quantify during which season adult individuals are more abundant in the shallow water column of the reefs’ matrices. To our knowledge, our study is the first based on a collection of nearly two thousand specimens of *Schindleria* and provides first baseline information on putative temporal distribution patterns worldwide, as well as year-round abundances in the Red Sea.

## Methods

### Sampling and data

**Figure 1:**
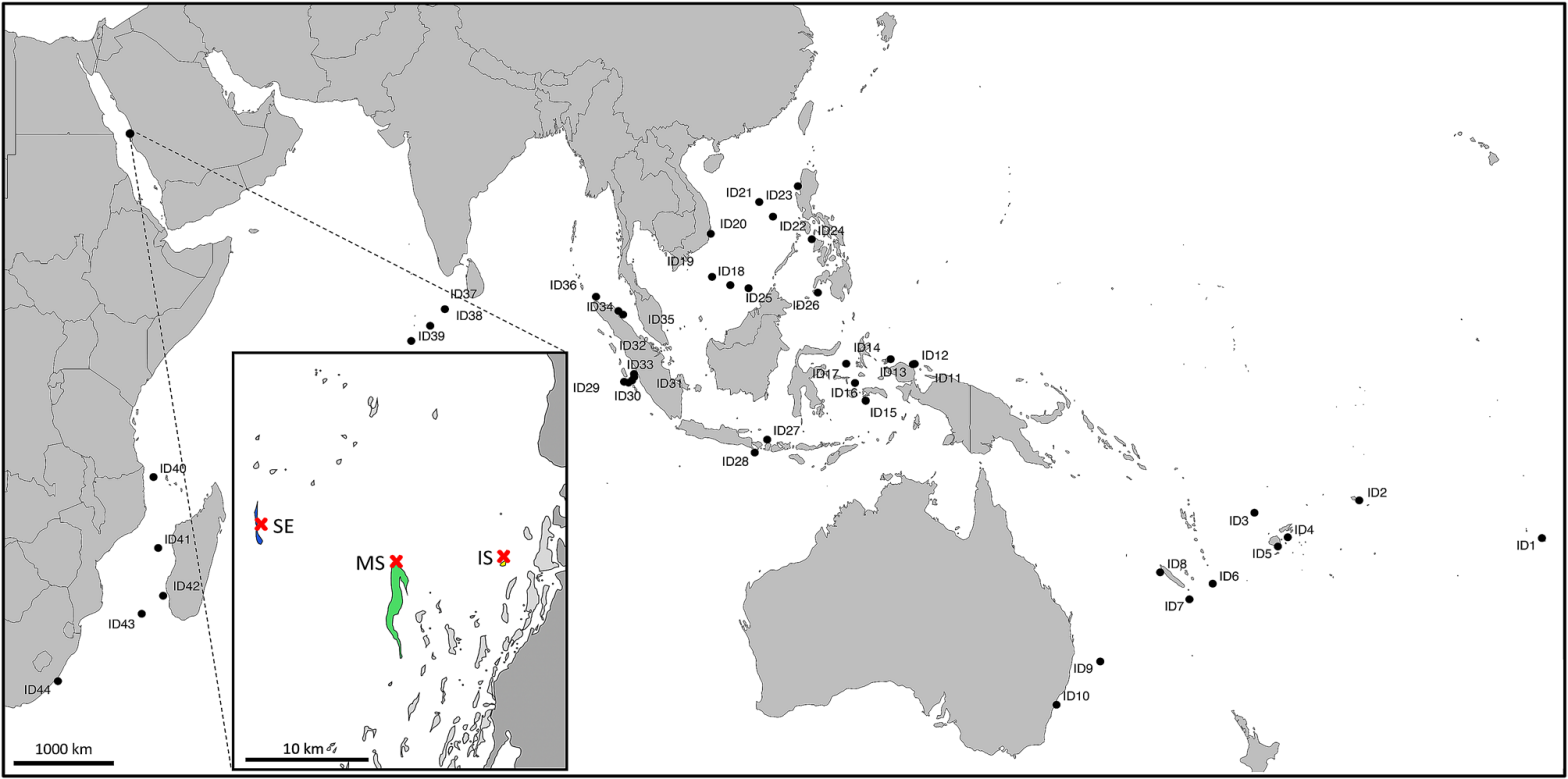
Collection sites of adult long dorsal fin (LDF) *Schindleria:* from the DANA Expedition 1928–1930 (all sites represented by a “dot”: ID1–ID44, following Ahnelt and Sauberer^12^) and from the Red Sea (with a zoomed-in section displaying the three reefs where collection took place using a LED powered light trap along a cross-shelf gradient from the shore to the shelf edge, near the coast of Thuwal, Saudi Arabia). Coral reefs within the zoomed-in Red Sea section are given in “light grey” while the land sections are in “dark grey” and the sampled reef sites are color coded, with the “yellow” reef inshore (IS), the “green” reef at the mid-shelf (MS), and the “blue” reef at the shelf edge (SE). The “red” crosses indicate the location of deployment of the LED powered light traps for this study. The map was generated in R v. 4.0.2 (https://www.r-project.org) using the libraries “sp”, “maps”, “maptools”, “mapproj”, “mapdata”, “ggthemes” and “ggplot2”.

Adult LDF *Schindleria* samples (i.e., females with visibly developed ovocytes and males with distinctive urogenital papillae) were collected at three reefs: one reef inshore “IS”, one at the mid-shelf “MS”, and one at the shelf-edge “SE” off of the coast of Thuwal, Red Sea, Saudi Arabia (blue, green, and yellow reef, respectively, in the zoomed map section of Fig. 1), using a set of three replicate light traps per reef (i.e., nine light traps total) attached to fixed moorings positioning the light trap ~ 2 m below the surface, at the northern end of the wave-protected (eastern) side of each reef, with a bottom depth of approx. 8 m to 14 m. Light traps are efficient sampling tools that use light to attract fish larvae (and other pelagic invertebrates and larvae) during the night time. The light traps were deployed for approximately 24 h for collections to take place during the entire night time. Prior to each sampling/deployment, the rechargeable batteries of the light traps were replaced with fully charged ones (batteries lasted over 24 h, but had reduced power) and each light trap and mooring were cleaned to assure the same starting conditions for each sampling and to avoid sampling biases due to algal growth, emerging chemical cues, or differing light intensity. For all qualitative and quantitative analyses, the catches from all three light traps were pooled per reef for each sampling date. The pooled samples are currently stored (as a loan) in the genetics laboratory of the ichthyology department of the Natural History Museum of Vienna, Austria. This research was undertaken in accordance with the policies and procedures of the King Abdullah University of Science and Technology (KAUST). Permits for sampling in Saudi Arabian waters were obtained from the relevant Saudi Arabian authorities. The study did not involve live specimens or endangered or protected species. KAUST implemented its Institutional Animal Care and Use Committee in December 2016; the collections in this paper were concluded prior to that date.

### Correlations of abundances of adult LDF *Schindleria* with lunar phases

The collapsible LED light traps (Bellamare 500-micron mesh) were first deployed for 32 consecutive days (from the 16th October to the 16th November 2014) to capture variation in abundance related to lunar phases (within a complete lunar month). The daily light trap samples were assigned to one of the four main moon phases depending on the date of their collection relative to the lunar day: the new moon “NM” (comprising the 26th–29th and the 1st–4th lunar day), the 1st quarter “1stQ” (from the 5th–11th lunar day), the full moon “FM” (from the 12th-19th lunar day), and the 3rd quarter “3rdQ” (from the 20th-25th lunar day).

To further assess the importance of moon phases on the abundance of *Schindleria* on a global scale, data on adult LDF *Schindleria* abundances from sampling stations from the DANA Expedition 1928–1930 (“ID1–ID44”, mainly sampled with S 150 and S 200 open stramin-nets, following Ahnelt and Sauberer^12^, where all available metadata on the samples can be found) were taken and categorized into the four main moon phases in the same way as the Red Sea data. Historical moon phases were taken from “moongiant.com”. The “moon phase” was then used as the explanatory variable (i.e., as a factor) for the abundance of adult LDF *Schindleria* (a) from the DANA Expedition and (b) from the Red Sea in two separate linear regression models (i.e., generalized linear models in R, *glm* function, R Core Team^30^). Due to overdispersion observed in both data sets (evaluated by using the dispersion test in the *AER* R package^31,32^) we used the quasi-poisson family distribution in the *glm* function to fit the model. An ANOVA was then performed between the null-model and our alternative model for each of the two data sets. The resulting (alternative) linear regression models from each data set were then compared to one another. Herefore, we plotted the log_10_ coefficients of the linear regressions of the abundances in the different moon phases of both datasets and assessed the significance of the differences of abundances of adult LDF *Schindleria* among the different moon phases, and whether these changes in abundances were similar between the two data sets. To compare the slopes of the linear regression models of the two datasets, the moon phases were converted to numeric ranks following the light intensity of each moon phase for which the “NM” was given the value “0”, the FM the value “2” and the 1st and 3rd quarter moons (1stQ and 3rdQ) the value of “1”, since both of them represent the value of one illuminated quarter of the moon. The *glm* function was again applied using the quasi-poisson family to correct for overdispersion of the data; and the respective slopes of the models of each dataset (Red Sea vs. DANA) were plotted to check for significant differences in the correlations between moon phases and abundances of adult LDF *Schindleria*.

As a last step to examine potential relations of adult LDF *Schindleria* catches with moon phases, the age of a subset of 44 specimens collected around the new moons (from May, June, and July 2015, from the upcoming section, preserved in 95% EtOH) at the three Red Sea locationswere assessed by counting daily growth increments of the sagittal otoliths. The specimens were sexed and measured (SL, standard length) before the extraction of right and left sagittal otoliths under an Olympus SZ61 stereomicroscope equipped with a polarized light filter. The pair of otoliths was completely immersed in epoxy resin and fixed to a microscopy slide. The otoliths were photographed under a Zeiss AXIO Lab.A1A microscope equipped with an ATRAY camera. No further preparation was needed to reveal daily increments. Additionally, the pictures taken from the otoliths for the counts of microincrements were categorized with a qualitative scale (C1 – C3), where C1 had poor resolution, were too damaged and/or in a state that made the readings of microincrements impossible, and were thus discarded from the analysis; C2 had tissue remains or were broken but provided enough resolution to assess numbers of microincrements; and C3 were in ideal condition. The otoliths were counted independently at least three times by the same reader (V.R.) using the photographs of the otoliths, which did not reveal any information on the sampling date or location, in order to obtain unbiased estimates of the age of each sample and its hatch date. Normality of the data was tested using the Shapiro–Wilk test. Counts of the right and/or the left sagittae were used, because both counts did not differ significantly (Wilcoxon-test, *z* = 0.30, *P* = 0.76). Therefore, in cases where one of the two sagittae otoliths were broken or too blurry to measure, the other available otolith was used; to calculate the median age. The hatch frequencies over the lunar cycle were determined by back-calculating the hatch dates from the sampling dates (using the median ages of the specimens) to determine the “Lunar hatch day” (LHD) and transform the lunar cycle to an angle variable. The lunar cycle (of 29 days) was converted to a 360° cycle, setting the new moon as day 1. Thereby, LHD = the number of days since the new moon*(360/29). A uniform distribution around the lunar cycle was tested using Rao’s spacing test^33^ as it is a robust circular statistical test able to analyze bi- and multimodal distributions. All statistical analyses related to otolith increments were performed using the free software Past 4.0^34^.

### Relationships of abundances of adult LDF *Schindleria* with temporal and spatial conditions

Using the same sampling procedure, sites, and moorings previously mentioned, light traps were deployed during five consecutive nights around the new moon for twelve consecutive months (from February 2015 to January 2016) to assess variation in abundance related to seasonal environmental (i.e., temporal) conditions and habitat/site-related (i.e., spatial) differences among the reefs within the Red Sea (i.e., the “IS”, the “MS”, and the “SE” reef, Fig. 1). Sea surface temperatures (SST) at each reef were further recorded during this period with an acoustic doppler current profiler (ADCP, Nortek AS, data available in Appendix S3^35^) to define seasons. The light traps also attracted larval fish recruits of which the abundances were spatio-temporally regulated by environmental conditions^35^. Hence, in addition to temperature, we included the abundances of coral reef fish larvae collected by the light traps as a predictive variable for the abundance of adult LDF *Schindleria.* The abundance of larval coral reef fishes likely mirrors a range of ecosystem and environmental parameters/conditions and was therefore considered as a good proxy to represent changes in the environment and the habitat composition. Additionally, physiologically and morphologically, adult LDF *Schindleria* strongly resemble fish larvae and frequently both co-occur in ichthyoplankton samples, for which the relation between the abundance of both may also be interconnected, as they may on the one hand profit from similar environmental conditions (i.e., increasing food availability) but on the other hand pose higher competition for similar resources (i.e., prey and shelter), with the benefit of a putatively “diluted” predation risk. Altogether, the local abundances of larval coral reef fish (as total counts), the sampling location (as reef site), the SST (in °C), and the month of sampling were considered in the initial model to perform linear regression on the abundances of adult LDF *Schindleria*. The best fitting model was selected by the AIC using the *step* function starting from the full-model (i.e., “backward”) in R.

## Results

### Sampling and data

A total of 1996 adult LDF *Schindleria* specimens were collected in the LED light traps deployed in the central Red Sea, Thuwal, Saudi Arabia, during this study.

From these, 204 specimens were collected during the daily sampling efforts of the lunar month from the 16th October to the 16th November (2014), for the assessment of abundances in relation to moon phases. The other 1792 specimens were caught during the twelve consecutive new moon collections from February 2015 to January 2016 for the yearlong spatio-temporal assessment of abundances. From the latter, the daily growth increments of the sagittal otoliths of 41 mature LDF specimens were successfully read for age determination, the estimation of hatch dates, and the assessment of lunar reproductive cycles.

Additionally, data on the temporal abundance of 502 adult LDF specimens from the DANA Expedition was extracted from Ahnelt and Sauberer^12^ to assess putative worldwide trends in abundances of adult LDF *Schindleria* with moon phases (Table 1).

**Table 1:** Total abundances of adult LDF *Schindleria* (All) observed in each of the four main moon phases: the new moon (NM), the 1st quarter moon (1stQ), the full moon (FM), and the 3rd quarter moon (3rdQ) comprising two different data sets: The Red Sea collections (at an inshore (IS: 22.3055° N, 39.0493° E), a mid-shelf (MS: 22.3054° N, 38.9684° E), and a shelf-edge (SE: 22.3415° N, 38.8538° E) reef) and the collection from the DANA Expedition from 1928–1930 (DANA)^12^.

Collection	Moon Phase			
	*NM*	*1stQ*	*FM*	*3rdQ*
***All***	200	139	35	332
***DANA***	116	97	15	274
***Red Sea***	84	42	20	58
*Inshore* (IS)	2	12	10	0
*Mid-shelf* (MS)	60	30	9	29
*Shelf-edge* (SE)	22	0	1	29

### Abundances of adult LDF *Schindleria* related to lunar phases

**Figure 2:**
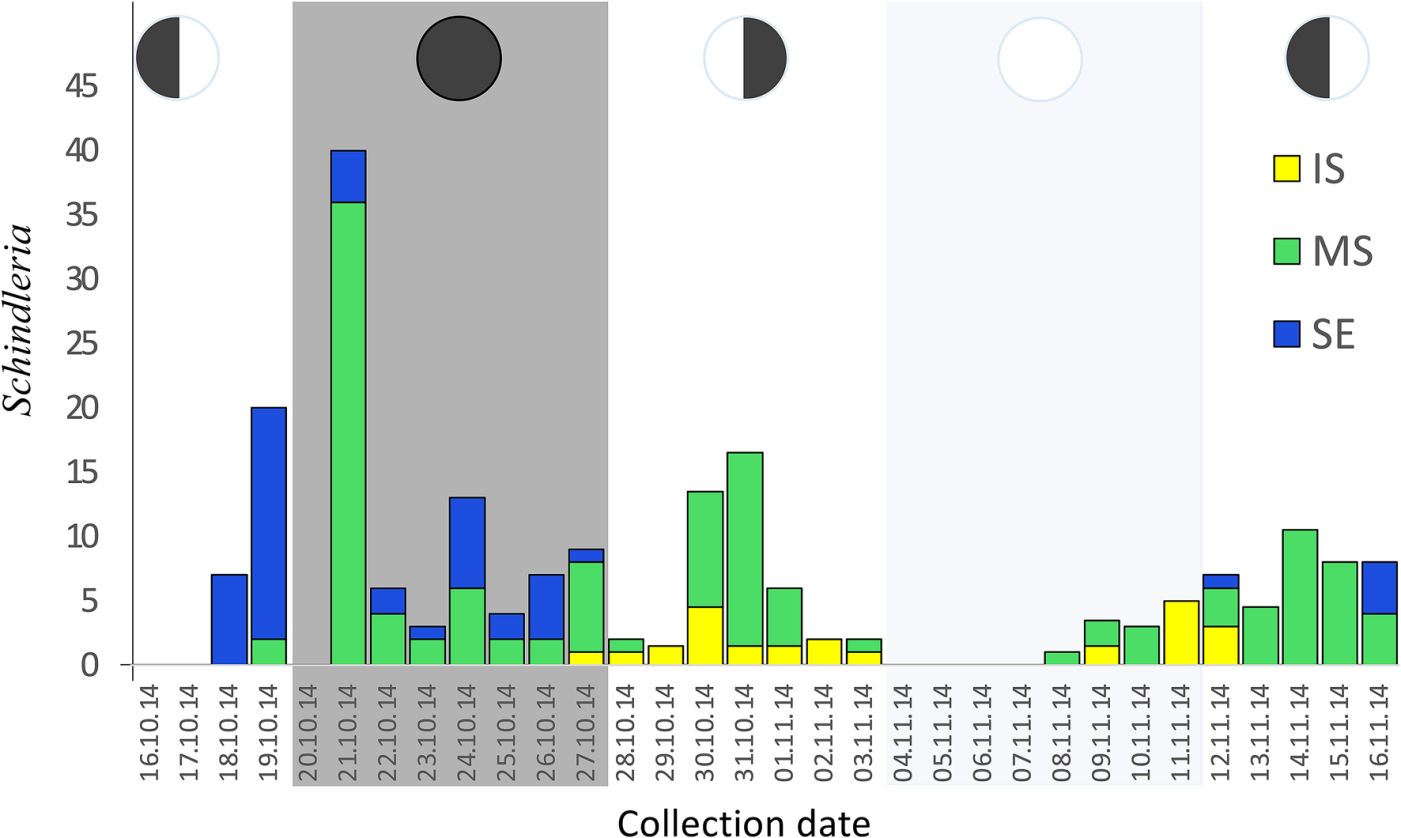
Barplot representing the total abundances of adult LDF *Schindleria* collected during one lunar cycle in the Red Sea of Saudi Arabia. The abundances of specimens (y-axis) are color-coded per sampling locations: from an inshore reef (IS) in “yellow”, from a reef at the mid-shelf (MS) in “green”, and from one at the shelf-edge (SE) in “blue”. Collections took place using three replicate LED powered light traps (collapsible Bellamare model) at each sampling location for the period of 32 consecutive nights, from the 16th October to the 16th November (2014; x-axis). Different moon phases are indicated with pie charts on the top of each shaded section of the plot starting with the 3rd quarter moon, followed by the new moon, the 1st quarter moon, the full moon, and ending again with the 3rd quarter moon (from left to right).

Out of the 204 adult specimens collected daily during a lunar month (Oct-Nov, 2014) in the Red Sea, only 4% were caught during the days around the full moon (± 3 days), while more than 40% were caught around the new moon (± 3 days) (see Fig. 2). Similarly, during the DANA Expedition, only 3% of the 502 adult LDF *Schindleria* specimens were caught during days around the full moon (± 3 days), while more than 33% were caught during days around the new moon (± 3 days). In both cases there was a tenfold increase in adult LDF *Schindleria* from full moon to new moon catches (Table 1).

**Figure 3:**
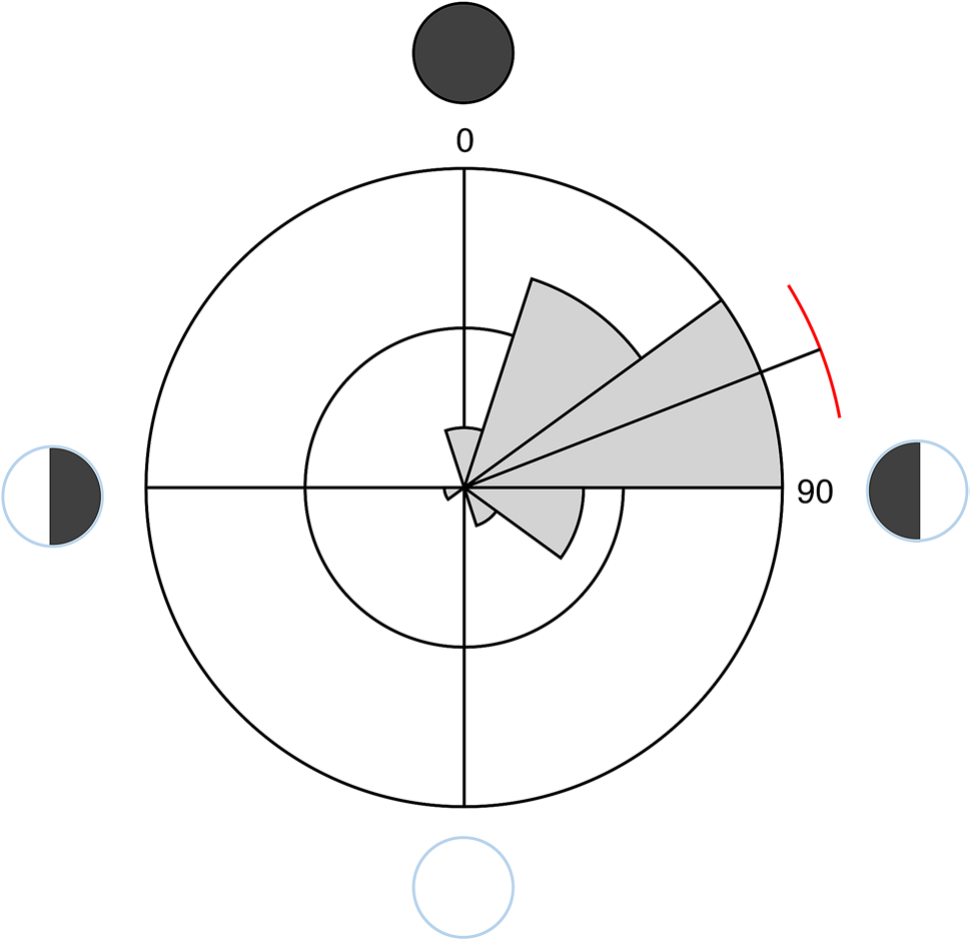
Distribution of hatch frequencies of LDF *Schindleria* from the Red Sea along a lunar cycle (indicated with the four different black and white pie charts). The new moon corresponds to 0° (top), the 1st quarter moon to 90° (right), the full moon to 180° (bottom), and the 3rd quarter moon to 270° (left). The “black” line gives the angular mean and the “red” arc the angular variance of the data and the pie sections within the circle, the number of hatch events during those lunar days.

**Figure 4:**
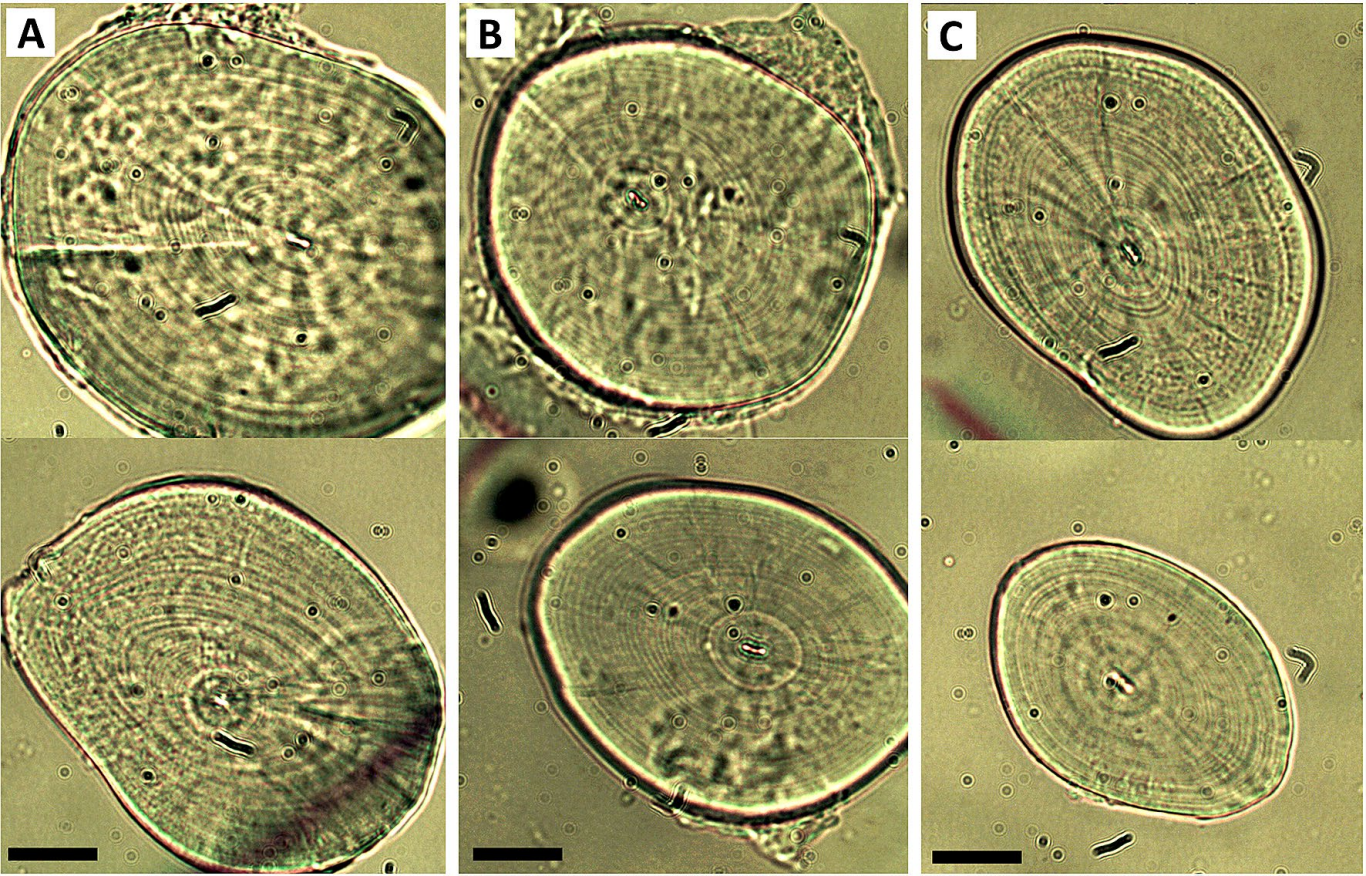
Sagittal otoliths from mature *Schindleria* specimens sampled with light traps from an inshore (**A**), a mid-shelf (**B**), and a shelf-edge (**C**) reef in the Red Sea, near the coast of Thuwal, Saudi Arabia. These otoliths represent otoliths of ideal conditions (i.e., “C3” category) to count daily growth increments and yielded similar average numbers of increments (between 26 and 28) despite slightly differing shapes and sizes. All otoliths are to scale, with the bar at the bottom (left) of each panel representing 20 μm. The otolith at the top of each panel represents a rounder and larger otolith while the otolith at the bottom a more oval-shaped and smaller otolith.

Otolith counts revealed the median age of mature LDF *Schindleria* specimens from the Red Sea to be 27 days (± 1 days MAD; Table 2). Moreover, through the back-calculation of the hatch dates using the age of the specimens and the collection date, we found a significant hatch pattern with lunar periodicity (Rao’s spacing test, *P* < 0.00), for which the angular mean was found ca. 5 days after the new moon (circular mean = 68.8°; Fig. 3). Together, the abundances of adult LDF *Schindleria* and the derived hatching events indicated that adult LDF *Schindleria* may be approaching the reef around the new moon for reproduction after which a few days later the eggs hatch, the larvae grow, and the specimens mature to return to the reef matrix on time for the next new moon, from which their reproductive cycle starts over. The oldest three individuals in our samples were 30 days old and the youngest two were 22 days old, but did not represent the largest and smallest individuals in the collection (26.3 mm and 9.7 mm, respectively with an average of 16 mm). Females carried large eggs and their ovaries were relatively full. Males had well developed urogenital papillae and all specimens were within the expected size range of adults (Chi^2^: P = 0.99). It thus seems likely that LDF *Schindleria* is completing its life cycle (i.e., one generation time) within a single lunar cycle. Lastly, despite similar age, we found differences in the sizes and shapes of the otoliths (Fig. 4), which may be indicative of the presence of different LDF species among our samples^36^. Note that otolith readings by two other independent readers (V.M.-V. and Camilo Rodríguez-Valentino) also resulted in a significant hatch pattern with lunar periodicity (Rao’s spacing test, *P* < 0.01), but slightly increased the median age (30 days (± 3 days MAD); data not shown).

**Table 2:** Otolith data from female (F) and male (M) adult specimens (standard length “SL” in mm) of LDF *Schindleria* from the Red Sea, collected from May to July 2015 at three reefs along a cross-shelf gradient (one inshore “IN”, one at the mid-shelf “MS”, and one at the shelf-edge “SE”) near the coast of Thuwal, Saudi Arabia. The last columns “L” and “R” indicate the quality assigned (C1-C3) to each, left and right sagittal otolith, respectively, where C1 represents poor/unreadable conditions, C2 conditions sufficiently good for readings, and C3 ideal conditions.

**N**	Reef	Sex	SL(mm)	Sampling date	Hatch date	Age	L	R
1	IN	–	14.64	–	–	27	C3	–
2	IN	F	18.4	18/5/15	25/4/15	23	–	C1
3	IN	F	19.22	18/5/15	18/4/15	30	C3	C1
4	IN	F	17.93	18/5/15	21/4/15	27	C1	C1
5	IN	M	18.55	18/5/15	22/4/15	26	C1	C3
6	IN	M	15.59	18/5/15	20/4/15	28	C2	C3
7	IN	M	15.32	20/5/15	23/4/15	27	C2	C2
8	IN	F	11.96	21/5/15	21/4/15	30	C2	C2
9	SE	–	20.48	18/6/15	25/5/15	24	C2	C3
10	SE	–	14.2	18/6/15	–	–	–	–
11	SE	–	13.86	18/7/15	7/7/15	11	–	C2
12	SE	F	14.68	18/7/15	24/6/15	24	C2	C3
13	SE	F	14.37	18/7/15	22/6/15	26	C3	C3
14	SE	F	14.83	18/7/15	23/6/15	25	C3	–
15	SE	F	16.09	18/7/15	20/6/15	28	C2	C2
16	SE	M	14.63	19/7/15	21/6/15	28	C2	C1
17	MS	F	16.08	17/6/15	20/5/15	28	C3	C2
18	MS	F	17.05	17/6/15	20/5/15	28	C3	C2
19	MS	F	16.9	17/6/15	21/5/15	27	C2	C1
20	MS	M	16.13	17/6/15	20/5/15	28	C3	C2
21	MS	M	13.89	17/6/15	23/5/15	25	C2	–
22	MS	M	15.52	17/6/15	22/5/15	26	–	C2
23	MS	M	14.8	17/6/15	24/5/15	24	C2	C2
24	MS	M	16.28	17/6/15	18/5/15	30	C2	C2
25	MS	M	10.4	17/6/15	–	–	C2	C2
26	MS	M	26.25	19/6/15	27/5/15	23	C2	C2
27	MS	F	11	19/6/15	28/5/15	22	–	C2
28	MS	F	17.63	19/6/15	22/5/15	28	C2	C1
29	MS	F	18.63	19/6/15	24/5/15	26	C2	C2
30	MS	M	16.83	19/6/15	26/5/15	24	C2	C2
31	MS	M	18.4	20/6/15	23/5/15	28	–	C1
32	MS	M	16.88	20/6/15	25/5/15	26	C2	C2
33	IN	M	11.19	19/6/15	–	–	C2	C2
34	IN	–	14.69	16/6/15	18/5/15	29	C2	–
35	IN	–	16.52	16/6/15	20/5/15	27	C2	C2
36	MS	F	17.54	16/6/15	21/5/15	26	C3	C2
37	MS	F	18.53	16/6/15	–	–	–	C1
38	MS	F	14.85	16/6/15	22/5/15	25	C3	C2
39	MS	F	14.87	16/6/15	20/5/15	27	C1	C2
40	MS	F	15.76	17/6/15	20/5/15	28	C2	C2
41	SE	M	9.67	18/7/15	22/6/15	26	C1	C3
42	SE	M	14.19	18/7/15	21/6/15	27	C3	–
43	SE	F	16.71	18/7/15	20/6/15	28	C2	C2
44	SE	F	15.67	18/7/15	21/6/15	27	–	C2

**Figure 5:**
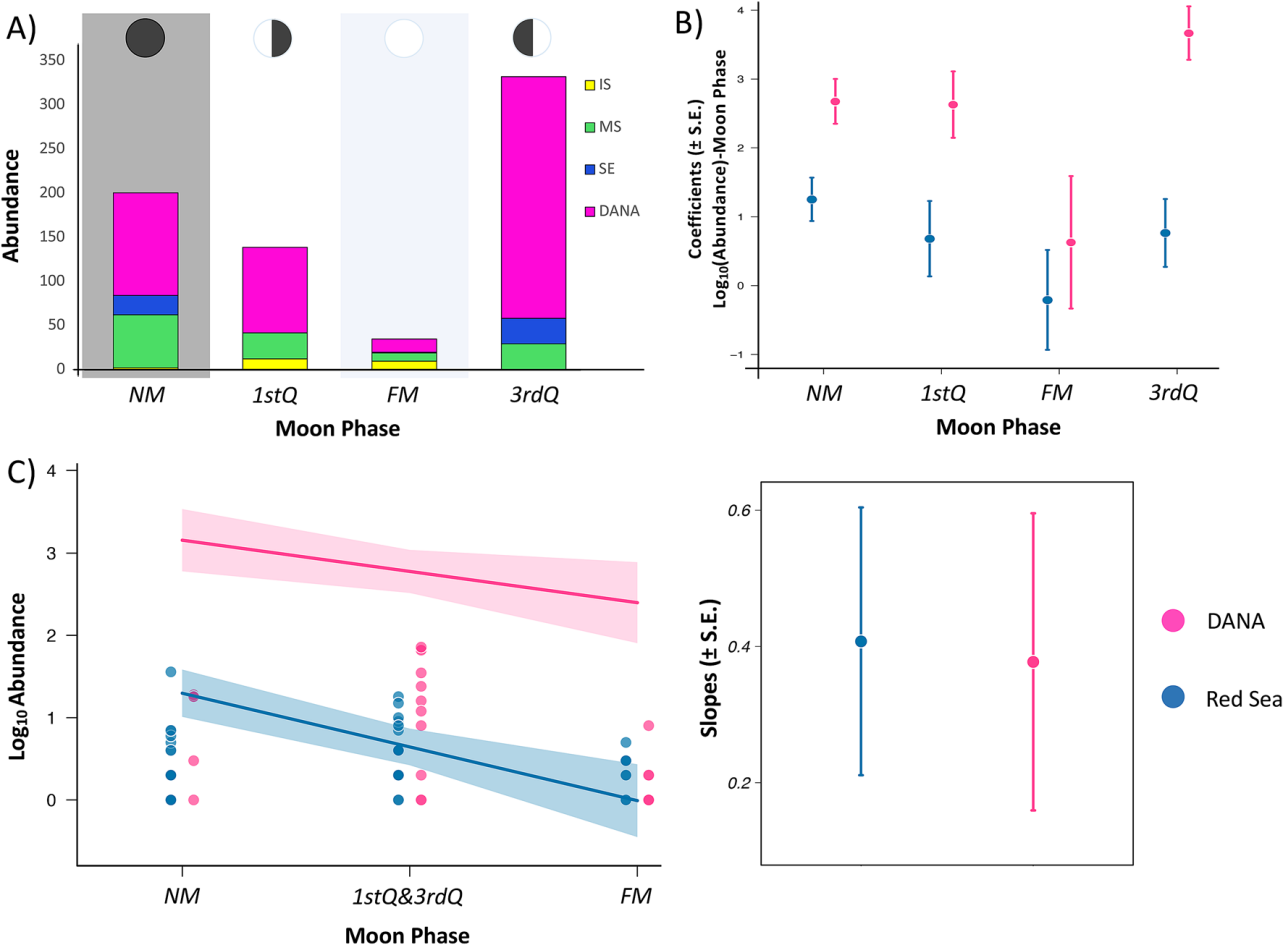
Relations between abundances of adult LDF *Schindleria* and the four main moon phases (the new moon “NM”, the 1st quarter “1stQ”, the full moon “FM”, and the 3rd quarter “3rdQ”) in two different datasets: one from an Indo-Pacific collection from the DANA Expedition 1928–1930 (DANA) and the other from a Red Sea collection (from the 16th Oct to the 16th Nov, 2014). Values from the DANA Expedition are always color-coded in “pink”. Panel A displays the abundances in the Red Sea color-coded per sampling site: inshore/IS reef in “yellow”, mid-shelf/MS reef in “green”, and shelf-edge/SE reef in “blue”. In Panel B and C, the Red Sea abundances are given as total values in “blue”. Panel A displays the total abundances (y-axis) for each dataset per moon phase (x-axis). The moon phases are represented by the circles on the top of the barplot and the brightest moon phase (FM) highlighted by a “light-blue” shaded box, while the darkest moon phase (NM) by a “black” shaded box. Panel B displays the coefficients of linear regression on the corrected Log_10_ Abundances (± SE; y-axis) of each moon phase (x-axis) of the two main datasets (DANA vs. Red Sea). Panel C displays, on the left plot, the abundances of each day of sampling (as dots) within a moon phase (on the x-axis; where the moon phases are grouped by light intensity for which the 1stQ and the 3rdQ moons are grouped in the middle of the regression) and on the right plot the respective slopes of the linear regression of both datasets of which the mean does not differ significantly between the two data sets. The lowest abundances of adult LDF *Schindleria* were generally during the FM.

From the linear regression models, in both datasets the alternative model fitted better than the null model. The differences in the abundances of adult LDF *Schindleria* in relation to the moon phases were most significant for the collections from the DANA Expedition, with the highest abundances caught in the 3rd quarter moon followed by the new moon; while in the Red Sea, it was the new moon, which yielded the highest abundances followed by the 3rd quarter moon (see Fig. 5A and B). However, in both datasets, the full moon yielded the lowest abundances, followed by the 1st quarter moon; and the differences between the full moon and the new moon abundances were always significant (i.e., in the linear regressions of both datasets; Fig. 5A and B). Additionally, the global trend of relations between lunar phases and abundances of adult LDF *Schindleria* was supported by the slope of the generalized linear models of both datasets, which were similar and of which the means did not significantly differ (Fig. 5C).

### Spatio-temporal abundances of adult LDF *Schindleria* within the Red Sea

**Figure 6:**
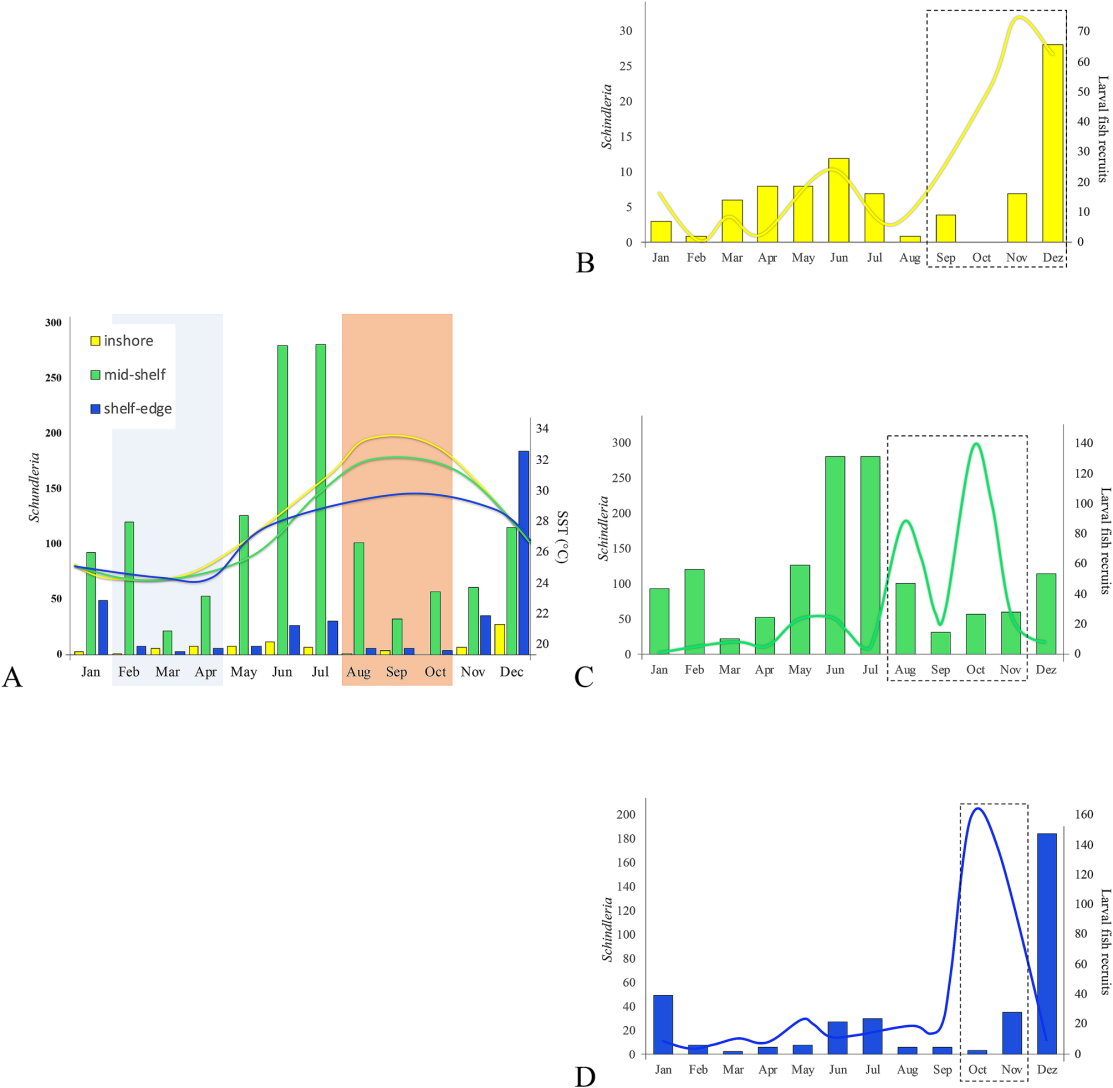
Barplot of the year-round abundances of adult LDF *Schindleria* (y-axis) from the Red Sea (Thuwal, Saudi Arabia) caught with LED powered light traps (monthly, during five days around the new moon, from February 2015 to January 2016) at three different reefs along a cross-shelf gradient: one inshore (“yellow”), one at the mid-shelf (“green”), and one at the shelf edge (“blue”). Panel A: adult LDF *Schindleria* abundances in relation to sea surface temperatures (SST, in °C). The total number of individuals for each sampling site collected each month is given as vertical bars (values correspond to the y-axis to the left). Temperature (SST) profiles per reef are also color-coded and given as a continuous line (values correspond to y-axis to the right). A “light-blue” shaded box indicates the months with temperature minima (February to April) and an “orange” shaded box indicates the months with temperature maxima (August to October), representing the hottest and coldest seasons, following Robitzch et al.^35^. Peaks of abundances are observed outside both boxes. Panel (**B**) to (**D**): adult LDF *Schindleria* abundances in relation to the abundance of larval fish recruits from light trap collections^35^, color-coded per reef site as in Panel (**A**). The total number of adult LDF *Schindleria* individuals collected at each sampling site is given by vertical lines and their values correspond to the left y-axis. Profiles of the abundances of larval fish recruits are given by continuous lines and their scale is given by the right y-axis. Dashed boxes indicate the months with peaks in recruitment of coral reef fish larvae (sensu Robitzch et al.^35^). Peaks of abundances of adult LDF *Schindleria* do not generally coincide with peaks of larval fish recruitment.

The mid-shelf reef site near the coast of Thuwal, Saudi Arabia, had the highest abundances of adult LDF *Schindleria* among all our Red Sea sites. Seventy-five percent (1341 specimens) of the total catches were collected at this site, followed by 366 specimens collected at the shelf-edge reef, and only 85 at the inshore reef (Fig. 6). Plotting the year-round abundances of adult LDF *Schindleria* for all sites and their respective sea surface temperature (SST), it was evident that the highest abundances of adult LDF *Schindleria* were neither during the hottest season of the year, nor during the coolest, but rather in between those two seasonal temperature extremes (Fig. 6A). Additionally, in relation to the presence of larval coral fishes, peaks in abundances of adult LDF *Schindleria* were also temporally decoupled from the peaks of abundances in coral reef fish recruitment (Fig. 6B,C).

Linear regression models were used to corroborate these observations. Initial models of the abundances of adult LDF *Schindleria* were created for the entire data set (i.e., SST, number of larval fish recruits, reef site, and month as explanatory variables). However, the best fitting model only included the reef site as the most significant explanatory parameter, having a relative importance of 0.62 among all variables of the initial model (which explained 65.2% of the total variance). Hence, linear regression models on the abundance of adult LDF *Schindleria* were also performed per reef site. At the inshore reef, the initial model including SST and the number of larval fish recruits was the best fitting model and was able to explain 43.9% of the total variance of the data with a relative importance of 0.77 for SST, and of 0.24 for the number of larval coral reef fish recruits. Contrastingly, the same initial model did not fit well neither at the mid-shelf reef nor at shelf-edge reef. In both latter cases, only very little of the variance was explained by this model (14.8% and 2.9%, respectively) and the number of larval fish recruits was relatively more important (0.67—0.99) compared to SST (0.001—0.33).

## Discussion

*Schindleria,* the smallest coral reef fish in the world, potentially has the largest populations and the fastest generation times of any reef associated fish, supported by our findings; and its high turnover rates may have a substantial ecological role in reef ecosystems. We, hypothesized that adult LDF *Schindleria* may exhibit periodic migrations from the pelagic into the reef matrix coupled with lunar cycles as part of their reproductive behavior. We used a large collection of nearly two-thousand adult LDF *Schindleria* specimens from the Red Sea and data from a recently published collection of more than five hundred adult LDF *Schindleria* from the DANA Expedition in 1928–1930^12^ in order to support our hypothesis and access more information on the biology and reproductive behavior of this fish.

In our study, we found evidence for global trends in the abundances of adult LDF *Schindleria* related to lunar cycles. We further found local seasonal and spatial differences in abundances potentially related to differing ecological and environmental conditions among the sampled reefs in the central Red Sea. Based on our results, we propose that adult LDF *Schindleria* at coral reefs are most abundant during environmentally moderate conditions (i.e., not the hottest seasons in the Red Sea, nor those with peaks in coral reef fish recruitment) and during the darker moon phases, since these tiny paedomorphic fishes will rather emerge and/or approach the reef matrix in the absence of light (from the moon) to protect themselves from nocturnal visual predators; and that the collection of primarily mature specimens at the reefs supports the idea that these periodic migrations may be related to reproductive cycles. Back-calculations of hatch dates from the Red Sea specimens based on otolith microincrements corroborated that LDF *Schindleria* larvae hatched few days after the adult abundance peak, further supporting our idea. Moreover, our results suggest that the reproductive cycle and one generation time of LDF *Schindleria* may be completed within a single lunar month, resulting in the fastest generation times among coral reef fishes. In the following, we discuss in detail our two major findings: the global trend of low abundances of adult LDF *Schindleria* around the full moon and the generally high year-round abundances of adult LDF *Schindleria,* yet decreased during seasonal temperature extremes and recruitment peaks of larval coral reef fishes in the Red Sea^35^.

The Red Sea collection and that from the DANA Expedition had significant differences in the abundance of adult LDF *Schindleria* in relation to moon phases. Adult LDF *Schindleria* were generally absent in catches during the full moon, while peaks of abundances were during the 3rd quarter (i.e., collections from DANA Expedition) and the new moon (i.e., Red Sea collections), with a tenfold increase compared to full moon catches, in both collections. All specimens from the Red Sea were mature adults and females carried large eggs, suggesting that these high abundances during darker moon phases may be timed aggregations for reproduction, conveniently avoiding high predation risks at night by the many visual predators of coral reefs.

Although *Schindleria* has been suggested to be a lagoon completer within the coral reef matrix^15^, rising evidence for the presence of a substantial number of adult LDF *Schindleria* far away from coral reefs (between 320 to 360 km) or in deeper pelagic waters (up to 1000 m depth)^12^ indicate a strong association of LDF *Schindleria* with the pelagic environment, at least during a portion of their life-cycle, which is further supported by our findings. *Schindleria* are very short-lived (max. age of 42 to 60 days^3,9^), probably beating the record of the dwarf goby *Eviota sigillata*^37^; and reach maturity at an extraordinarily early age for a vertebrate (23 days^3^). In our study the median age of the mature LDF adults was of 27 (± 1 MAD) days, which is close to the total length of a lunar cycle (of 29 days); and the hatch dates of these Red Sea specimens were significantly occurring around similar lunar days, shortly after the new moon. Hence, we propose that LDF *Schindleria* does not reside within the reef matrix as a lagoon completer, but that it completes the pelagic larval duration in the course of one single lunar cycle in order to reach progenic maturation right before migrating back to aggregate in the shallower coral reef waters for reproduction, when adults are most abundant. For instance, Isari et al.^38^ found *Schindleria* within plankton tows in the Red Sea, taken from pelagic waters during days prior and around the full moon close to our sampling sites; but they mainly captured juvenile and larval *Schindleria* (S. Isari, pers. comm.), also suggesting that the spatio-temporal abundances and distributions of *Schindleria* may differ with ontogeny. Similarly, we observed a slight shift in lunar phase with the highest abundance of adult LDF *Schindleria* between the two collections studied (i.e., 3rd quarter moon in the DANA Expedition vs. the new moon in the Red Sea). This could also be explained by the exact location of the sampling. As in Isari et al.^38^, the DANA specimens were not collected directly in the reef matrix (as were our Red Sea specimens) but from more pelagic waters, and the sampling method used during the DANA Expedition were plankton tows, which do not actively attract specimens within the reef-matrix (as do the light traps used during the Red Sea sampling) but capture those specimens present in the water column. Hence, while we were able to collect only mature specimens that were already at the reef for reproduction, the DANA Expedition may have collected adult specimens that were yet in the pelagic, on their way to the reef, but still a couple of days away from reaching complete maturity. Due to the preservation method used in the DANA collection (i.e., the samples had initially been placed in a formalin/ethanol mixture), the otolith structures were dissolved, precluding the estimation of age. Nonetheless, we hypothesize that the trends at other geographic locations will likely resemble results from the Red Sea, where mature LDF adults only aggregate periodically at the reefs during the new moon. Other recent collection efforts using light traps at the shores of Rapa Nui/Easter Island, Chile, and in the Western Indian Ocean, also indicate higher presence of mature *Schindleria* during the new moon (V. Robitzch, unpubl. data). It remains unclear why *Schindleria* evolved such a short generation time^39^, but our results suggest that coupling that with the length of a lunar cycle may be a strategy that works on a global scale.

The observations from the inshore reef in the Red Sea were different and had a peak in adult LDF *Schindleria* abundances during unexpected moon phases: the 1st quarter and the full moon. However, this site had overall an extremely low abundance of *Schindleria*, limiting the value of interpretation of these result; for which we cannot say whether these abundances reflect biological patterns/trends or strayed individuals or the result of other factors such as light intensity of the surroundings vs. the light trap, habitat alterations, turbidity, distance to the pelagic environment, current regimes, etc. For example, the inshore sampling site is very close to the coastal town of Thuwal and the King Abdullah University of Science and Technology, which are both sources of noise and light at night. Sound has proven to be negatively related to the abundance of *Schindleria*^36^. Specimens from the inshore site could have been generally more attracted by the stronger coastal lights than our light trap and predation risk may also be increased in the stronger illuminated inshore reefs^36^. Additionally, our data likely includes observations of multiple species, as already indicated by the differences in the macrostructures of the otoliths we extracted; for which outliers may also be representative of differences among species and their specific biological and ecological features.

Lastly, among all the sampling sites within the Red Sea, we further concluded that the highest abundances of adult LDF *Schindleria* were neither found during seasonal temperature extremes (i.e., maxima and minima) nor during the peaks of coral reef fish recruitment. Most specimens were caught outside of the hottest season, which can be related to the extraordinarily high sea surface temperatures of the Red Sea^35,38,40^ and may not hold true outside the Red Sea. More interestingly, peaks in abundance of adult LDF *Schindleria*, although outside the seasons with highest temperatures, did not coincide with the peaks of abundance of recruiting coral reef fishes in the Red Sea, which is also outside of the hottest season^35,38^. *Schindleria*’s larvae are likely benefitting from the same conditions as are other coral reef fish larvae^35^; but the temporal differences between the peaks of abundance of *Schindleria* and of other coral fish larvae may be explained by the longevity and biology of *Schindleria.* As opposed to longer-lived coral reef fishes, *Schindleria* cannot restrict reproduction or the termination of their PLD to the most favorable season. The species’ longevity demands monthly, year-round reproduction^41,42^; and *Schindleria* may have a more successful PLD outside the periods of peaks in coral reef fish reproduction, since it will likely have less competition for resources, particularly within the extremely oligotrophic central Red Sea. The mortality of larval stages of *Schindleria* may also increase during abundance peaks of coral reef fish larvae as these may prey on tiny *Schindleria* larvae leaving fewer adults to complete the PLD and return to the reefs. Additionally, due to the morphological similarity between adult *Schindleria* and fish larvae, both may have similar predators. Hence, the abundance of *Schindleria* may be seasonally reduced during periods of recruitment peaks of coral reef fish larvae because of associated higher abundances of predators adapted to feed on incoming larval recruiting cohorts. Altogether, we argue that *Schindleria*’s unique life history and short generation time forces it to start a new generation every month “despite the weather”, which is why it is generally abundant year-round. Nonetheless, *Schindleria* is yet a species-poor genus, likely due to the lack of data rather than the lack of biodiversity; and a large number of cryptic species comprising this taxon^36^ will likely reveal additional species-specific behaviors, distributions, and trends.

## Data Availability

All otolith pictures are available from the authors upon request.

## References

[1] Giltay L (1934). Les larves de Schindler sont-elles des Hemirhamphidae?. Notes Ichthyol. Mus. Roy. d’Hist. Nat Belgique.

[2] Johnson GD, Brothers EB (1993). *Schindleria*: a paedomorphic goby (Teleostei: Gobioidei). Bull. Mar. Sci..

[3] Kon T, Yoshino T (2002). Diversity and evolution of life histories of gobioid fishes from the viewpoint of heterochrony. Mar. Freshw. Res..

[4] Randall, J. E. & Cea, A. *Shore fishes of Easter Island*. (University of Hawaii Press, 2011).

[5] Kon T, Yoshino T, Mukai T, Nishida M (2007). DNA sequences identify numerous cryptic species of the vertebrate: a lesson from the gobioid fish *Schindleria*. Mol. Phylogenet. Evol..

[6] Robitzch V, Schröder M, Ahnelt H (2021). Morphometrics reveal inter- and intraspecific sexual dimorphisms in two Hawaiian Schindleria, the long dorsal finned *S. praematura* and the short dorsal finned *S. pietschmanni*. Zool. Anz..

[7] Schindler O (1930). Ein neuer *Hemirhamphus* aus dem Pazifischen Ozean. Anzeiger der Akad. der Wissenschaften Wien.

[8] Schindler, O. Sexually mature larval Hemiramphidae from the Hawaiian Islands. *Bull. Bernice P. Bish. Museum* 1–28 (1932).

[9] Landaeta MF, Veas R, Castro LR (2002). First record of the paedomorphic goby *Schindleria praematura*, Easter Island, South Pacific. J. Fish Biol..

[10] Watson W, Walker HJJ (2004). The world’s smallest vertebrate, *Schindleria brevipinguis*, a new paedomorphic species in the family Schindleriidae (Perciformes: Gobioidei). Rec. Aust. Museum.

[11] Kon T, Yoshino T, Nishida M (2010). Cryptic species of the gobioid paedomorphic genus *Schindleria* from Palau, Western Pacific Ocean. Ichthyol. Res..

[12] Ahnelt H, Sauberer M (2020). Deep-water, offshore, and new records of Schindler’s fishes, *Schindleria* (Teleostei, Gobiidae), from the Indo-west Pacific collected during the Dana-Expedition, 1928–1930. Zootaxa.

[13] Bruun AF (1940). A study of a collection of the fish *Schindleria* from South Pacific waters. Dana Rep..

[14] Jones S, Kumaran M (1964). On the fishes of the genus *Schindleria* (Giltay) from the Indian Ocean. J. Mar. Biol..

[15] Leis JM (1994). Coral Sea atoll lagoons: closed nurseries for the larvae of a few coral reef fishes. Bull. Mar. Sci..

[16] Belyanina TP (1989). Ichthyoplankton in the regions of the Nazca and Salas y Gomez submarine ridges. J. Ichthyol..

[17] Parin NV, Mironov AN, Nesis KN (1997). Biology of the Nazca and Salas y Gomez submarine ridges, an outpost of the Indo-West Pacific fauna in the Eastern Pacific Ocean: composition and distribution of the fauna, its communities and history. Adv. Mar. Biol..

[18] Ahnelt H, Sauberer M (2018). A new species of Schindler’s fish (Teleostei: Gobiidae: *Schindleria*) from the Malay archipelago (Southeast Asia), with notes on the caudal fin complex of *Schindleria*. Zootaxa.

[19] Leis JM, Goldman B, Read SE (1989). Epibenthic fish larvae in the Great Barrier Reef Lagoon near Lizard Island, Australia. Japanese J. Ichthyol..

[20] Thacker C, Grier H (2005). Unusual gonad structure in the paedomorphic teleost *Schindleria praematura* (Teleostei Gobioidei): a comparison with other gobioid fishes. J. Fish Biol..

[21] Young S-S, Chiu T-S (2000). New records of a paedomorphic fish *Schindleria praematura* (Pisces: Schindleriidae), from Waters of Taiwan. Acta Zool. Taiwanica.

[22] Watson, W. & Leis, J. M. *Ichthyoplankton of Kaneohe Bay, Hawaii. A one-year study of fish eggs and larvae*. 1–178 (University of Hawaiʻi Sea Grant Program, 1974).

[23] Leis, J. M. & Trnski, T. *The larvae of Indo-Pacific shorefishes*. (New South Wales Univ. Press, Sydney & Univ. of Hawaii Press, 1989).

[24] Fricke R, Abu El-Regal MA (2017). *Schindleria nigropunctata*, a new species of paedomorphic gobioid fish from the Red Sea (Teleostei: Schindleriidae). Mar. Biodivers..

[25] Fricke R, Abu El-Regal MA (2017). *Schindleria elongata*, a new species of paedomorphic gobioid from the Red Sea (Teleostei: Schindleriidae). J Fish Biol.

[26] Abu El-Regal MA, Kon T (2019). First record of the Schindler’s fish, *Schindleria praematura* (Actinopterygii: Perciformes: Schindleriidae), from the Red Sea. Acta Ichthyol. Piscat..

[27] EAbu El-Regal M, Kon T (2008). First record of the paedomorphic fish *Schindleria* (Gobioidei, Schindleriidae) from the Red Sea. J. Fish Biol..

[28] Ahnelt H (2019). Redescription of the paedomorphic goby *Schindleria nigropunctata* Fricke & El-Regal 2017 (Teleostei: Gobiidae) from the Red Sea. Zootaxa.

[29] Contreras JE, Landaeta MF, Plaza G, Ojeda FP, Bustos CA (2013). The contrasting hatching patterns and larval growth of two sympatric clingfishes inferred by otolith microstructure analysis. Mar. Freshw. Res..

[30] Team, R. C. R: a language and environment for statistical computing (version 3.6). https://www.R-project.org (2020).

[31] Kleiber, C. & Zeileis, A. *Applied econometrics with R.* (Springer Science & Business Media, 2008).

[32] Kleiber, C. & Zeileis, A. AER: applied econometrics with R. R package version 1.1. (2009).

[33] Batschelet, E. *Circular statistics in biology*. (Academic Press, New York, 1981).

[34] Hammer Ø, Harper DAT, Ryan PD (2001). PAST: paleontological statistics software package for education and data analysis. Palaeontol. Electron..

[35] Robitzch V, Berumen ML (2020). Recruitment of coral reef fishes along a cross-shelf gradient in the Red Sea peaks outside the hottest season. Coral Reefs.

[36] Whittle, A. G. *Ecology, abundance, diversity, and distribution of larval fishes and Schindleriidae (Teleostei: Gobioidei) at two sites on O’ahu, Hawai’i*. (University of Hawaiʻi, 2003).

[37] Depczynski M, Bellwood DR (2005). Shortest recorded vertebrate lifespan found in a coral reef fish. Curr. Biol..

[38] Isari S (2017). Exploring the larval fish community of the central Red Sea with an integrated morphological and molecular approach. PLoS ONE.

[39] Depczynski M, Bellwood DR (2006). Extremes, plasticity, and invariance in vertebrate life history traits: insights from coral reef fishes. Ecology.

[40] Nanninga GB, Saenz-Agudelo P, Zhan P, Hoteit I, Berumen ML (2015). Not finding Nemo: limited reef-scale retention in a coral reef fish. Coral Reefs.

[41] Hernaman V, Munday PL (2005). Life-history characteristics of coral reef gobies. I. Growth and life-span. Mar. Ecol. Prog. Ser..

[42] Lefèvre CD, Nash KL, González-Cabello A, Bellwood DR (2016). Consequences of extreme life history traits on population persistence: do short-lived gobies face demographic bottlenecks?. Coral Reefs.

